# Dough Rheology, Physico‐Chemical, and Sensory Properties of Bread From Wheat/Fresh Green Mature Banana Puree Composite

**DOI:** 10.1002/fsn3.72489

**Published:** 2026-10-19

**Authors:** Ediriisa Mugampoza, Norah Nankya, Arthur Tabula, Martin Mutambuka, Khadijah Nakyinsige

**Affiliations:** ^1^ Department of Food Science and Technology, Faculty of Science Kyambogo University Kyambogo Uganda; ^2^ Uganda National Bureau of Standards Kampala Uganda

**Keywords:** banana, bread, bread quality, dough rheology

## Abstract

Wondering whether fresh green mature bananas could be used for supplementation of resistant starch and dietary fiber in bakery products, our study set out to evaluate the effect of partial substitution of wheat flour with different forms of banana (fresh whole fingers, fresh pulp, whole finger flour, and pulp flour) on dough rheology, physico‐chemical and sensory properties of bread. A hybrid banana variety, N23 was selected for the study. Wheat‐banana composite (70:30%, w/w) bread was formulated using 210 g wheat flour, 90 g banana, 1000 mL water, 120 g sugar, 4.0 g yeast and 3.0 g salt and a control with 300 g wheat flour. The addition of fresh and/or powdered bananas significantly increased the time (*p* < 0.05) required to reach maximum consistency, reduced resistance to deformation (PrMax) but had no significant effect on consistograph properties and water absorption (*p* > 0.05). The control samples exhibited the lowest baking loss and the highest specific volume. Crust and crumb browning increased significantly with the addition of 30% bananas in comparison to the control. Resistant starch, dietary fiber and moisture content increased with increasing levels of banana substitution. Flavor and taste of bread decreased with the addition of banana but the average scores for general acceptability were moderate. Thus, in bread making, fresh banana or flour could be added up to 30% substitution levels with moderate effects on quality and consumer acceptability.

## Graphical abstract

The study determined the effect of partial substitution of wheat flour with different forms (fresh whole fingers, fresh pulp, whole finger flour, and pulp flour) of a hybrid banana variety, N23, on dough rheology, physico‐chemical, and sensory properties of bread. Wheat‐banana composite (70:30%, w/w) bread was formulated and used in the study.

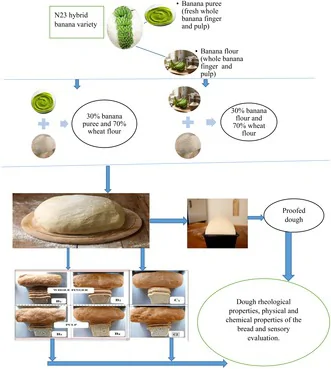

## 1 Introduction

Bread is one of the oldest and world's most widely consumed staple foods (Barnes 2025; Ribet et al. 2025). Bread has been commonly known as a fermented wheat‐based product made by mixing mainly wheat flour with water, yeast, and salt. The rationale for using wheat flour is due to its gluten content, which imparts desirable viscoelastic properties to the dough thereby contributing to the structure and texture of the final product (Chikpah et al. 2021; Kuktaite and Ravel 2020). During dough mixing, when water is added to wheat flour, gluten proteins develop a cohesive viscoelastic network that retains the gas generated during dough fermentation resulting in the formation of a leavened and fluffy bread crumb (Wieser et al. 2023; Delcour et al. 2012) making it difficult to produce superior quality bread without gluten (Chikpah et al. 2021).

Similar to other developing countries where the consumption of bread has continuously increased due to changing eating habits, steadily growing population, good taste and convenience (Noorfarahzilah et al. 2014), in Uganda, bread is mainly consumed as a breakfast snack by about 65% of the urban population and 31% of people in rural areas (Anna et al. 2014). The commonest limiting factor for bread consumption in Uganda is affordability. The high cost of bread is attributed to high production cost since wheat is imported from major European producers, which necessitates looking for alternatives from locally available materials. Using composite flours which combine wheat with other ingredients could diversify the flavor profile of the bread and save consumers from taste fatigue that is associated with consumption of the same type of bread made solely from wheat flour over an extended period (Cappelli and Cini 2021). United Nations' Food and Agriculture Organization (FAO) has also supported using composite flours from locally grown products as an alternative to wheat flour in food products if imports of wheat are to be reduced (Jisha et al. 2008).

Bananas are the world's fourth dietary staple food and most important agricultural commodity after corn, rice, and wheat (Sarawong et al. 2014; Subbaiah et al. 2013). Uganda is the highest producer of cooking bananas in Africa and ranks 2nd in the world after India (UBOS 2010). Uganda produces approximately 10.5 million metric tons of plantains, 0.5 million metric tons of dessert bananas, and 4.34 million metric tons of cooking bananas (FAO 2019). East African Highland cooking bananas are the main staple food in Uganda, contributing to food security in the country and grown on approximately 40% of the total arable land by 75% of the country's farmers (Ariho et al. 2015). However, much of this is marketed and distributed raw, with neither processing nor value addition, which limits its green life and increases its susceptibility to postharvest deterioration. Banana postharvest losses stand at an alarming 40% (Bezerra et al. 2013), thus, the need for innovations involving processing of fresh green mature bananas into value‐added products such as bread.

As observed by Petrontino et al. (2025), diversifying raw material sources in the bakery supply chain often faces technological limitations; lack of gluten is the limiting factor for the utilization of banana flour as a major baking ingredient. However, wheat/banana composite flour has successfully been used in bread formulation (Rahman et al. 2021; Muranga et al. 2010; Juarez‐Garcia et al. 2006), including the inclusion of peels in the flour (Gomes et al. 2016; Garzón et al. 2011). This study focused on using fresh green mature banana puree without drying as this would save the resources and energy used to dry the bananas. Although white bread is often preferred for its palatability, the consumption of whole‐wheat bread is promoted for its health benefits and/or reduced risk for disease and mortality (Gómez et al. 2020). Partial substitution of wheat with fresh green mature banana puree would yield products similar to whole‐wheat bread. Petrontino et al. (2025) pointed out that enrichment of wheat with alternative matrices represents a promising approach to produce bread with their high fiber content, high‐quality proteins, and enhanced health benefits.

Green mature bananas contain high levels of starch (22%) and low levels of natural sugars (1%), proteins (less than 1%), and lipids (0.15%–0.58%) (Gafuma 2018; Hapi et al. 2007). Gafuma (2018) highlighted that fresh green mature cooking bananas in Uganda are high in starch (4%–15.2%; of which 38%–68% is resistant starch). Moreover, the banana peel largely contains fiber and could therefore be a potential source of dietary fiber in functional breads. Resistant starch has attracted a lot of research interest because of its positive effects in the human colon and improvements on health (Fuentes‐Zaragoza et al. 2011). Similarly, dietary fiber is resistant to digestion and absorption in the small intestine, with complete or partial fermentation in the gut. It is a suitable substrate for bacterial fermentation in the colon, which helps to maintain its structure and proper function (Drzikova et al. 2005). High dietary fiber intake has been indicated to improve physiological and metabolic functions of the body (Drzikova et al. 2005; Champ and Guillon 2000). Together with resistant starch, dietary fiber also provides a favorable environment for growth of intestinal flora and increases intestinal microbial fermentation, thereby enhancing colon health. Thus, partial substitution of wheat with fresh green mature bananas would not only reduce the production cost of bread but also improve its functional properties such as boosting its resistant starch III and dietary fiber content. This study assessed the effect of using the different forms of raw fresh green mature N23 banana puree on dough rheology, resistant starch and dietary fiber content, and physico‐chemical and sensory properties of the banana‐wheat composite bread. The uniqueness of this study lies in introducing the utilization of raw banana puree in dough formulation, which eliminates the resources spent on the drying process.

## 2 Materials and Methods

### 2.1 Sample Collection

A bunch of fresh green mature N23; a hybrid variety of cooking bananas (Figure 1) was selected for the study. The agronomic characteristics of N23 are similar to those of the high‐yielding local variety, *Mpologoma*. When East African Highland Cooking Bananas (*Musa* spp.) locally known as *Matooke* became susceptible to a number of pests and diseases, the National Banana Research Programme at National Agricultural Research Organization (NARO) resorted to developing new cooking banana hybrids with increased resistance to drought, pests, and diseases. Unfortunately, most of the developed cooking banana hybrids faced rejection by the local end users because of a lack of sensory attributes considered significant for *Matooke* consumer acceptability. This makes industrial application or utilization of hybrid cooking banana varieties in baking necessary not only to reduce the production costs of bread since most of the wheat used in Uganda is imported but also to reduce the postharvest losses.

**FIGURE 1:**
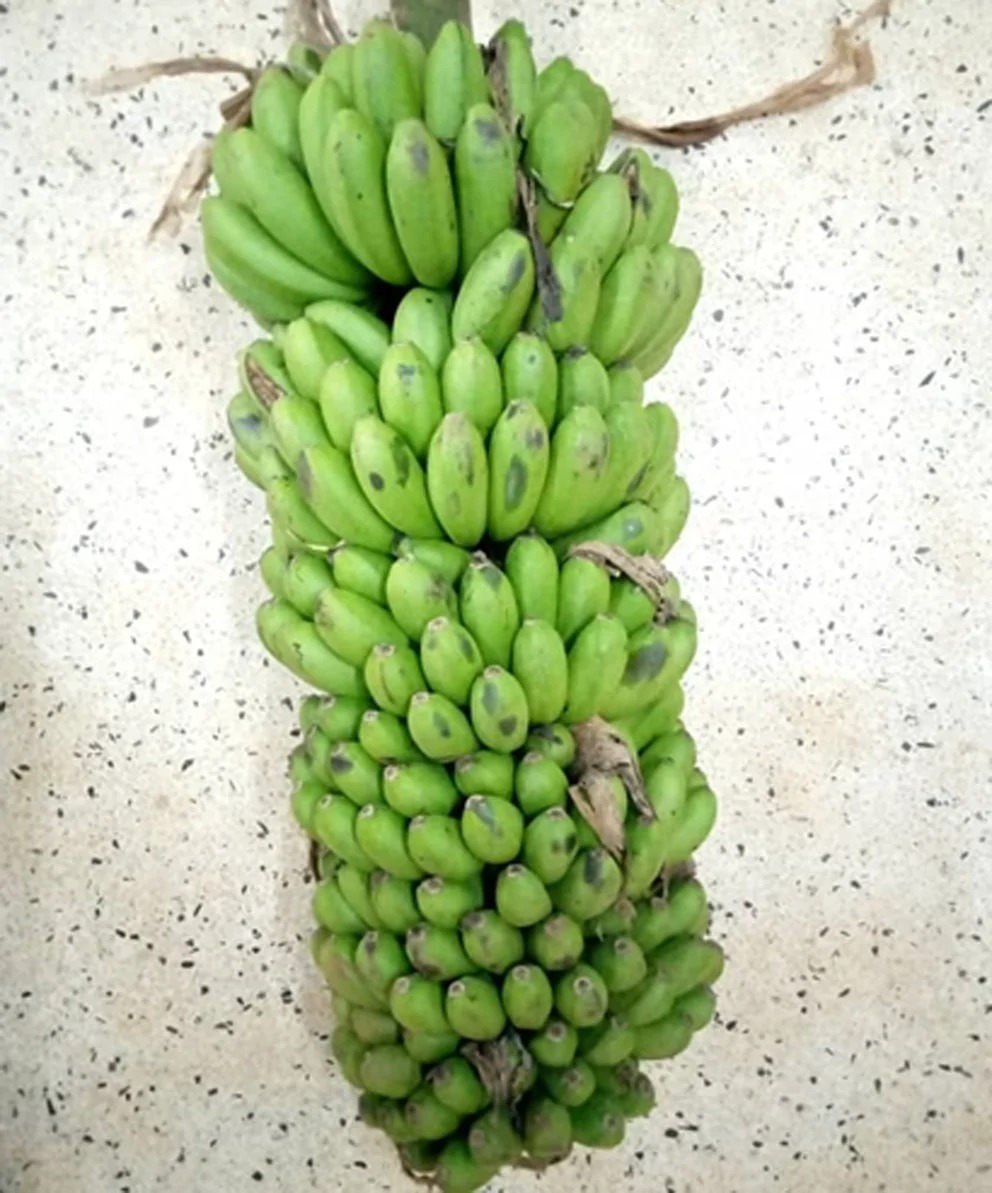
N23 hybrid banana variety used in the study.

The samples were obtained from NARO, Kawanda. The bananas were harvested at the green maturity stage (14 months from the time of planting), de‐clustered and immediately transported in iceboxes to the Kyambogo University Food Science Laboratory. At the Laboratory, they were sorted according to finger size and washed with potable water. The bananas were subdivided into four treatments, that is, Fresh Whole Banana Fingers (T1), Fresh Banana Pulp (T2), Whole Banana Finger Flour (T3), and Banana Pulp Flour (T4). Other ingredients, including Wheat flour (Azam home baking flour), Active dry yeast (Saf‐levure), shortening (Baker's pride), sugar (Kakira white sugar), and salt (Habari iodated edible salt), were obtained from Capital Shoppers, a local supermarket in Kampala city.

### 2.2 Preparation of Banana Flour

Banana flour was prepared according to the procedure described by Gomes et al. (2016). Samples from T2 and T4 were sliced to 5 cm thickness using a stainless‐steel knife and soaked in a solution of 1 g/100 mL citric acid for 5 min to prevent enzymatic browning. The slices were placed on a tray and dried in a conventional air oven (M120, Macadams, South Africa) at 60°C for 24 h. The dried samples were ground into fine flour using a blender (4696, Yutai, China) at 20,000 rpm and sieved through a 3 mm screen. The flour was packed in stomacher bags, sealed using an impulse sealer (IS400 Packer, China), and stored in a black polythene bag at ambient temperature (25°C) until use.

### 2.3 Formulation of Banana‐Wheat Composite Dough

Banana bread was prepared using banana—wheat composite flour mixture as shown in Table 1. The substitution rate for wheat flour (30%, w/w) was adopted from Manano et al. (2021) as the optimal inclusion level. The amount of water added to each formulation was adjusted based on the moisture content of the fresh banana to account for the water contributed by the puree and ensure a consistent total water content across formulations.

**TABLE 1:** Formulations of banana‐wheat composite bread dough.

Treatment	Wheat (g)	Bananas (g)	Water (mL)	Sugar (g)	Yeast (g)	Salt (g)	DM
T2	210	90	930	120	4.0	3.0	23.85
T1	210	90	930	120	4.0	3.0	25.54
T3	210	90	1000	120	4.0	3.0	ND
T4	210	90	1000	120	4.0	3.0	ND
C1	300	0.00	1000	120	4.0	3.0	ND
C2	150	150	1000	120	4.0	3.0	ND

Abbreviations: C_1_: 100% Wheat flour; C_2_: 50:50 (Banana flour: Wheat flour); ND: not determined; T1: Fresh whole banana fingers; T2: Fresh banana pulp; T3: Whole banana finger flour; T4: Banana pulp flour.

### 2.4 Determination of Dough Rheological Properties

#### 2.4.1 Consistograph Characteristics

Dough consistency behavior was studied following the Official International Association for Cereal Science and Technology method no. 173 (2011). Moisture content of the sample was determined using the air oven method after 24 h baking following procedures outlined in AOAC Official Method 925.10. The amount of the water added for hydration of the sample based on its moisture content was automatically calculated. Then, the mixer hook was inserted into the mixing bowl of the consistograph (model 2/2, serial no: 181, Chopin Technologies, France) followed by 250 g of the sample. Mixing of the flour sample ensued while the water‐dosing unit of the consistograph started adding water automatically. Mixing proceeded for 5 min and the consistograph simultaneously generated a consistographic curve and accompanying dough rheology properties, providing data about the dough's consistency and development. The water absorption capacity (WAC) and maximum pressure (PrMax) were determined.

#### 2.4.2 Alveograph Properties

The indices for dough resistance to extension (*P*), dough extensibility (*G*), and dough/baking strength (*W*) were determined using a Chopin alveograph (AlveoLAB, Chopin Technologies, Villeneuve‐la‐Garenne, France) according to the Grains and Cereals Association method no. 54–30. The alveograph characteristics included maximum over‐pressure (*P*) needed to blow the dough bubble, the average abscissa (*G*) at bubble rupture, the deformation energy (*W*) which relates to the surface under the curve indicating the necessary work input needed to inflate the dough, and the *P*/*L* ratio. The kneader hook was inserted into the mixing bowel of the alveograph followed by the dough sample. Kneading of the dough started while water was added automatically, and the process proceeded for 8 min. After, the laminated dough with uniform thickness was cut into pellets (5 × 5 cm). The pellets were placed in the resting chamber for 15 min and thereafter subjected to alveographic analysis to obtain five different curves following the standard Chopin+ protocol at 80 rpm mixing speed. The five curves were: initial equilibrium at 30°C, 8 min; heating to 90°C, 7 min (at a rate of 4°C/min); holding at 90°C for 7 min; cooling to 50°C, 5 min (at a rate of 4°C/min); and holding at 50°C for 5 min. Of the five curves obtained, only three most similar curves were selected, from which an average curve and accompanying rheology properties were generated.

### 2.5 Baking of the Composite Bread

The composite bread was baked using the straight‐dough Grains and Cereals Association method no. 10‐09 as described by Manano et al. (2021). The ingredients in Table 1 were weighed using a digital weighing scale (ATY224R, Shimadzu) and mixed with the required amount of water in a Macadams spiral dough mixer (model: SM 201S/1917220822) for 15 min. After mixing, 500 g dough portions were rounded, fermented (knocked back) and molded into a loaf shape, which was placed in a lightly greased bread tin. The tin was placed in a proofer (model: MP01‐05906‐05/19) for 30 min at 40°C and 80% relative humidity. The proofed dough was baked in a preheated Macadams rotary oven (model: M120) at 200°C for 20 min. The controls included dough prepared from wheat flour alone and wheat: fresh banana pulp (50:50) prepared simultaneously under similar conditions. After bread cooled for 24 h, placed in low‐density polyethylene bag and analyzed for physical, chemical and sensory properties.

### 2.6 Determination of Physical Properties of the Bread

#### 2.6.1 Loaf Volume

The seed displacement technique outlined in Grains and Cereals Association method 10–05 was followed to measure the loaf volume. Millet seeds were used instead of rapeseeds because the former are readily available in Uganda and their size is generally similar to that of rapeseeds. The container was filled with millet grains and leveled using a meter‐ruler. The millet seeds were withdrawn from the container into the measuring cylinder and their volume recorded. Thereafter, bread was placed into the container followed with millet grains until fully covered and leveled again. The volume of the remaining millet grains displaced by the bread was measured using a measuring cylinder and was considered as the volume of the bread expressed in cubic millimeters.

#### 2.6.2 Determination of Loaf Weight and Baking Loss

Loaf weight was measured using a digital analytical balance (PA423, Ohaus, Switzerland) 1 h after the loaf was removed from the oven. Baking loss was determined by the difference between the weight of bread dough before baking and weight of baked bread taken 24 h after baking (Chikpah et al. 2021).

#### 2.6.3 Cross Sectional Area of the Loaf Slice

The measured length and width of the bread slice cut from the centre of the loaf were used to calculate cross sectional area as follows:

$$\text{Area}\;\left({\mathrm{cm}}^{2}\right)=\text{length}\times \text{width}$$

#### 2.6.4 Crumb and Crust Color

Crust and crumb color was measured using a Lovibond Tintometer (CR‐400 Series, Konica Minolta, Osaka, Japan). The bread sample cut into a 2 × 2 mm cross section was placed onto the optical glass. Measurements were taken for the degree of blackness, whiteness, greenness, redness, blueness and yellowness using the L*a*b* color notation. Where L* measures the lightness of the sample, the higher the value the lighter the sample; a* measures the red‐green color axis, where +a* is red and −a* is green; and b* measures the yellow‐blue axis, where +b* is yellow and −b* is blue. The lightness (L* value) of the crumb was used to indicate product quality.

#### 2.6.5 Analysis of Crumb Texture Profile

Bread crumb texture was determined after 24 h of baking a using a TVT Texture Analyzer (TVT6700, Perten, Australia) equipped with a 5 kg load according to Grains and Cereals Association method no. 74‐09 and following the procedure described by Chikpah et al. (2021). The calibrated TVT uses the double cycle compression mechanism to test the bread crumb properties, fitted with a stainless steel 25 mm diameter probe (P‐CY255). The bread loaf was accurately sliced using an electrical slicing machine (Gravity, S/N 4710, South Africa) to give slices of equal thickness. Three slices were compressed at the same time to produce a required thickness of 50 mm for a single test. Texture profile analysis was carried out using a cylindrical aluminum probe with 25 mm diameter. A two (2) cm thick bread slice was obtained from the centre of the loaf and subjected to double cycle of compression. Two measurements were taken on the slice crumb at pre‐selected locations and analyzed for the following parameters; cycle until count, test speed 1.0 mm/s, post‐test speed 2.0 mm/s and maximum deformation, 50%. The Texture Analyzer software was used to obtain results of maximum force at peak, area for the first and second peak and time taken to reach peaks for each measurement. Texture properties of the sample were calculated using the obtained data as follows; (1) hardness; maximum force at peak of the first compression, (2) cohesiveness; area during the second compression divided by area during first compression, (3) springiness; time in seconds to second compression divided by distance covered during first compression and, (4) chewability; hardness × cohesiveness × springiness.

### 2.7 Determination of Chemical Properties of the Bread

#### 2.7.1 Moisture Content

The moisture content was determined using the air oven method after 24 h of baking following procedures outlined in AOAC Official Method no. 925.10. A clean dish was placed in an oven (Memmert; UFP 550) at 105°C for 1 h. After cooling in a desiccator for 30 min, the dish was weighed using an analytical balance (PA423, Ohaus, Switzerland) and the weight recorded. Approximately 5 g of the bread sample was weighed into the dish, placed in the oven (Memmert; UFP 550) set at 105°C for 3 h until constant weight, and then cooled in a desiccator. The weight of the dish containing the dry sample was measured and recorded. The determined moisture content was the difference between the weight of the sample before and after drying and expressed as a percent of the original sample weight.

#### 2.7.2 Crude Dietary Fiber

Crude dietary fiber content was determined following ISO 5498: 1981. Approximately 3 g of the defatted test sample was weighed. About 200 mL of the sulfuric acid solution (0.3 mol/L) was added to the test sample. The solution was mixed and boiled to 100°C for 30 min. Approximately 50 mL of cold water was added and the insoluble residue was separated using a separating device. The residue was washed with 50 mL portions of hot water at 100°C. The washing was repeated until the filtrate substantially tested neutral to litmus paper. The insoluble residue was collected in the incineration dish and dried at 130°C ± 2°C. After drying, the residue was incinerated in the muffle furnace at 550°C ± 2°C to constant mass. The incineration dish was cooled to room temperature in the desiccator and weighed again.

#### 2.7.3 Resistant Starch Content

Resistant starch content was determined using the Megazyme resistant starch assay kit by the enzymatic hydrolysis procedure according to AOAC method 2002.02 and AACC method 32–40.01. Approximately 0.1 g bread sample weighed into a 15 mL Falcon tube and mixed with a 10 mL solution of 1 M Sodium Chloride/0.5 M Hydrochloric acid. Approximately 10 mg/mL alpha amylase was added to it. The flask was incubated at room temperature (25°C) for 40 min to hydrolyze digestible starch. The mixture was centrifuged (Model 5804R, Eppendorf, Germany) at 6000 rpm for 10 min at 4°C to obtain an insoluble sediment. Then, 3 mL of Tris maleate buffer (pH 6.4–8.0) was added and the sediment solubilized. The solubilized sediment was heated in a water bath at 90°C for 30 min to gelatinize the starch. The starch gel formed was homogenized and 120 μL of amyloglucosidase solution added and mixed thoroughly using a vortex mixer. The mixture was incubated at 60°C for 45 min. After centrifugation (4°C) at 6000 rpm for 10 min, the resulting supernatant was used for analysis of resistant starch content. The supernatant (0.5 mL) was measured into a test tube and 0.5 mL phenol added. Approximately 1 mL of distilled water and 1 mL conc. H_2_SO_4_ were added. The solution was kept for 10 min in the dark for color development. The absorbance was determined using a spectrophotometer (3047, Jenway, England) at 490 nm.

### 2.8 Sensory Evaluation

The panel was trained following ISO 8586:2023 and sensory evaluation was done according to ISO 6658:2017. A panel comprising 30 persons was used to assess the sensory characteristics of the bread samples. A nine‐point hedonic scale was used to score the different organoleptic properties of the bread including appearance, color, flavor, taste, texture and general acceptance of the presented bread samples where 1 was rated dislike extremely and 9 was like extremely.

### 2.9 Statistical Analysis

All analyses were performed in triplicate. All chemical data were standardized by conversion to dry weight basis. To ascertain the mean difference, statistical analysis was performed using one‐way analysis of variance (ANOVA) and Tukey's post hoc test using the Statistical Package for Social Scientists (SPSS) software version 24. Pairwise comparison of the data was performed using XLSTAT. Differences were considered significant at *p* < 0.05. The relationship between green mature banana addition, dough rheology and bread quality characteristics were evaluated by principal component analysis (PCA) using the Unscrambler software version.

## 3 Results

### 3.1 Rheological Properties of the Banana‐Wheat Composite Dough

Changes in consistograph properties of the dough are given in Figure 2T1, T2, T3, T4, C1. The addition of fresh and/or powdered bananas significantly increased the time (*p* < 0.05) required to reach maximum consistency. The amount of water (water absorption, WA) needed for optimum production of dough did not significantly differ among treatments (*p* > 0.05; Table 2). Water absorption (WA) indices and the pressure (PrMax) obtained in all curves involving banana composites were generally lower than those of the control (100% wheat flour). The whole finger and pulp flours were almost similar to their respective fresh samples as there were no significant differences in consistograph properties (*p* > 0.05). Noteworthy, addition of bananas to wheat flour reduced resistance to deformation (PrMax) (Table 2). This implies poor dough handling behavior and low dough tolerance in the fermentation stage for wheat‐banana composite dough.

**FIGURE 2:**
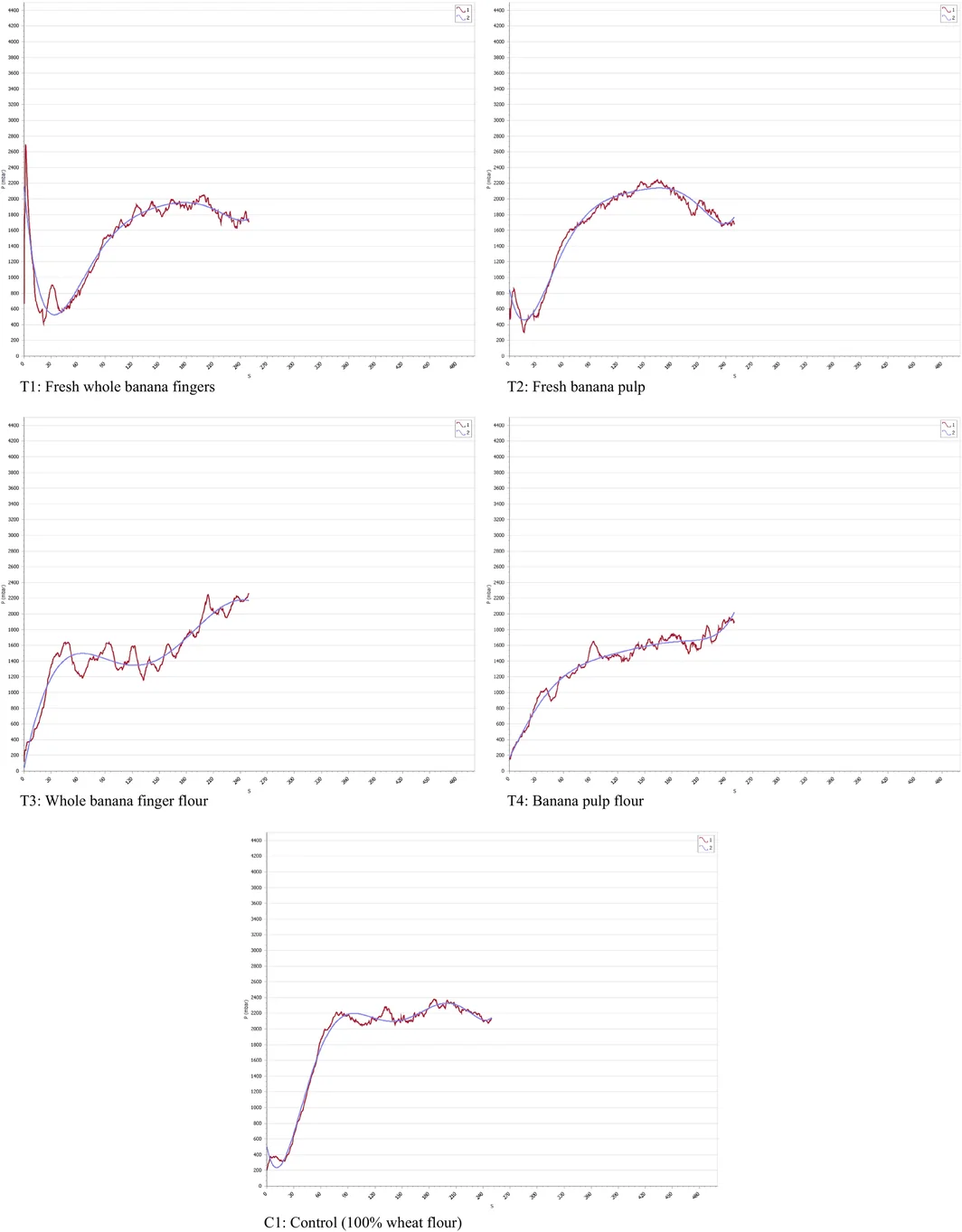
Changes in consistograph properties of dough obtained from wheat flour (70%) composited with 30% banana. T1: Fresh whole banana fingers. T2: Fresh banana pulp; T3: Whole banana finger flour; T4: Banana pulp flour; C1: Control (100% wheat flour).

**TABLE 2:** Rheological properties of dough obtained from wheat flour mixed with different forms of green mature cooking bananas.

Type of sample	Treatment	Alveograph properties				Consistograph properties	
		Configuration ratio	Elasticity index (%)	Baking strength (10^−4^ J)	Extensibility index (mm)	Water absorption (%)	PrMax (mbar)
Fresh	T1	1.60	10.2^b^	222^b^	95^b^	53.5^a^	2160^b^
	T2	1.40	10.7^b^	215^b^	102^a^	53.4^a^	2139^b^
Flour	T3	5.32	11.8^b^	109^bc^	19^c^	53.3^a^	2107^b^
	T4	6.00	12.3^b^	98^c^	17^c^	53.6^a^	2179^b^
Control	C1	1.40	54.1^a^	262^a^	67^b^	53.4^a^	2328^a^
	C2	1.14	15.9^b^	229^b^	123^a^	ND	ND

Abbreviations: C_1_: 100% Wheat flour; C_2_: 50:50 (Banana flour: Wheat flour); J: Joules; mbar: millibar; mm: millimeters; ND: not determined; T1: Fresh whole banana fingers; T2: Fresh banana pulp; T3: Whole banana finger flour; T4: Banana pulp flour.

Fresh whole finger (T1) and pulp banana (T2) samples had relatively similar alveograph profiles to that of wheat alone control (Figure 3T1, T2, T3, T4, C1). Generally, the flours had higher elasticity indices compared to fresh samples; hence, very low configuration ratios (*P*/*L*) and elasticity indices producing low baking strengths (Table 2). The elasticity indices for banana‐wheat composite samples ranged from 10.2 to 15.9, way lower than 54.1 of the 100% wheat samples. Overall, the elasticity indices of the composite mixtures were lower than that of the control, suggesting that the substitution level of bananas (30%) applied in the composite mixture was relatively high. Moreover, dough samples containing bananas exhibited lower baking strength and resistance to mechanical mixing compared to the control sample (Table 2), indicating that the former were weaker doughs.

**FIGURE 3:**
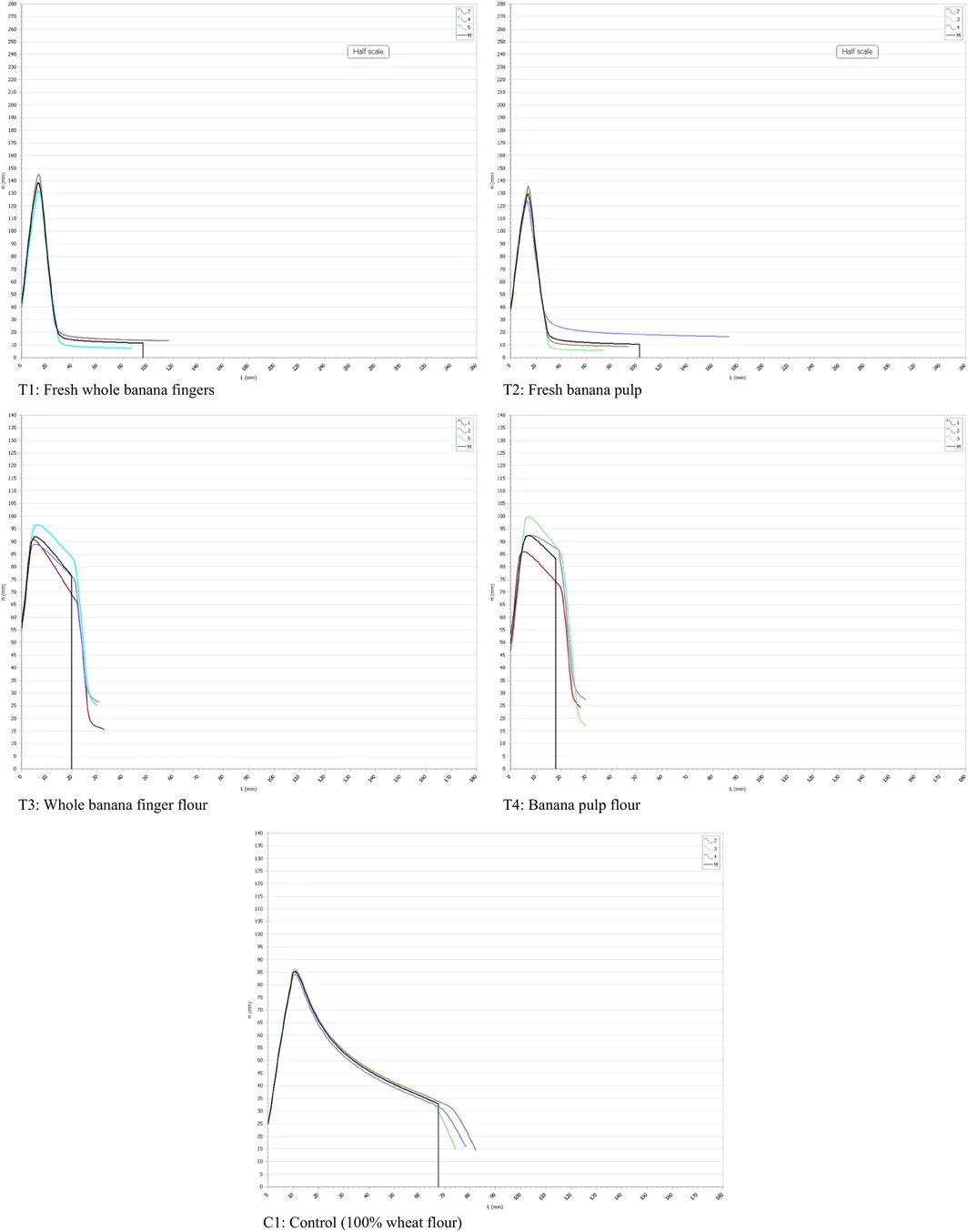
Changes in alveograph properties of dough obtained from wheat flour (70%) composited with 30% banana. T1: Fresh whole banana fingers. T2: Fresh banana pulp; T3: Whole banana finger flour; T4: Banana pulp flour; C1: Control (100% wheat flour).

### 3.2 Physical Characteristics of the Banana‐Wheat Composite Bread

The bread loaves obtained and their respective cross sections of the crumb are shown in Figure 4. Noteworthy, unlike rheological data which showed that fresh banana pulp and pulp flour at inclusion ratio of 30% produced weaker doughs, the general appearance of bread obtained from banana‐wheat composite doughs was appealing and comparable to that of the wheat alone bread. Flour samples generally had brighter crumb appearance. In fact, even when the fresh banana pulp inclusion level was raised to 50%, good quality bread was still obtained as shown in Figure 4C_2_. There was no observable difference in the crumb pore characteristics of fresh and flour samples. However, incorporation of whole banana fingers gave a duller crumb appearance compared to pulp.

**FIGURE 4:**
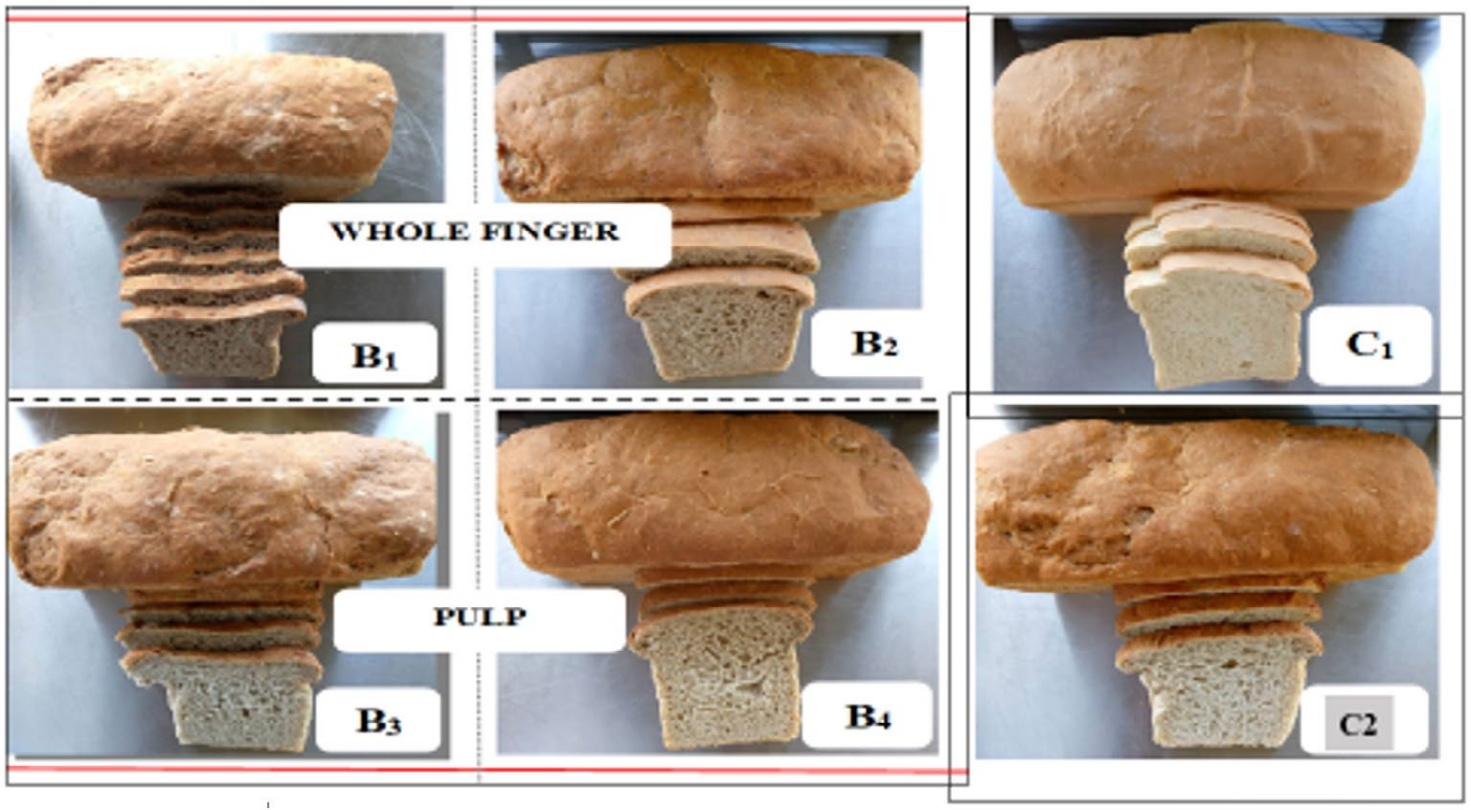
Image analysis of bread obtained from wheat flour (70%) composited with different forms. Fresh whole finger (B_1_), Whole finger flour (B_2_), Fresh pulp (B_3_) and Pulp flour (B_4_). Controls: 100% wheat flour (C_1_) and 50% Wheat: 50% N23 fresh pulp (C_3_).

Results of bread volumes obtained from the banana‐wheat composite mixture are shown in Table 3. Baking loss was highest in the 50:50% banana: wheat composite bread (C1) and lowest in the 100% wheat alone control sample (C2). The inclusion of banana in dough formulation had no significant effect on the cross sectional area of the bread (*p* > 0.5). The cross sectional area ranged from 85.76 cm^2^ to 119.23 cm^2^ with the sample containing the control (100% wheat) having the highest while that formulated from banana pulp flour (T4) had the lowest (Table 3). Loaf weight ranged from 530.50 g to 579.00 g. There was significant difference in the loaf weight of all formulations (*p* < 0.05). The volume of the control (100% wheat) bread was significantly higher than that of the samples containing bananas (*p* < 0.05). For bread containing banana, samples formulated from whole fresh fingers (T4) had the lowest loaf volume. Crust and crumb browning increased significantly with the addition of 30% bananas in comparison to the 100% wheat alone control (*p* < 0.05).

**TABLE 3:** Physical properties of bread obtained from wheat flour mixed with different forms of green mature cooking bananas.

Type of sample	Formulation	Baking loss	Cross sectional area (cm^2^)	Loaf weight (g)	Loaf volume (cm^3^)	Color of crumb/crust					
						L Crust	*a*	*b*	L Crumb	*a*	*b*
Fresh	T1	9.25^c^	109.02^a^	579.00^a^	1305.72^c^	23.75^ab^	−0.40^a^	29.70^ab^	22.15^b^	−7.25^ab^	27.00^b^
	T2	13.63^b^	85.85^a^	577.50^a^	1410.00^ab^	20.00^b^	−1.40^ab^	26.60^b^	24.25^b^	−7.60^ab^	29.55^ab^
Pulp	T3	7.97^d^	115.67^a^	571.00^a^	1310.26^c^	21.45^bc^	−0.30^a^	27.30^b^	24.30^b^	−5.45^a^	32.20^a^
	T4	14.91^ab^	85.76^a^	544.00^ab^	1410.00^ab^	23.95^ab^	−0.95^a^	30.05^ab^	24.85^b^	−8.25^b^	27.45^b^
Control	C1	7.71^c^	119.23^a^	570.00^a^	1439.33^a^	27.85^a^	−3.20^c^	32.40^a^	32.45^a^	−6.00^a^	35.65^a^
	C2	18.00^a^	94.87^a^	530.50^ab^	1360.13^b^	21.65^b^	−1.70^ab^	26.70^b^	23.95^b^	−7.55^ab^	28.40^ab^

*Note:* Values in columns with similar superscript letters are not significantly different at *p* > 0.05. Values are means of two independent replicates.

Abbreviations: C_1_: 100% Wheat flour; C_2_: 50:50 (Banana flour: Wheat flour); T1: Fresh whole banana fingers; T2: Fresh banana pulp; T3: Whole banana finger flour; T4: Banana pulp flour.

### 3.3 Chemical Characteristics of the Banana‐Wheat Composite Bread

Results for moisture, resistant starch, and dietary fiber content of bread from wheat flour mixed with different forms of green mature cooking bananas are shown in Table 4. Moisture content varied among samples, with 100% wheat samples (C1) exhibiting a significantly lower content than banana—wheat composites (*p* < 0.05). Samples with fresh whole banana fingers (T1) had the highest moisture content. A similar trend in moisture content was observed among the banana flour samples. Resistant starch content ranged from 1.08 g/100 g in 100% wheat bread to 2.21 g/100 g in the 50% wheat flour: 50% banana flour bread (*p* < 0.05; Table 4). In general, resistant starch significantly increased with increasing the level of banana substitution. Dietary fiber was highest (1.95%) in bread made from fresh whole fingers (T1) and lowest in the 100% wheat bread (C1). Additionally, bread formulated from whole bananas had higher dietary fiber content than one made from banana pulp. Fresh whole fingers had the highest contribution to dietary fiber content of bread compared to all other treatments. In fact, increasing the proportion of banana pulp to 50% did not pose a significant increase in the fiber content of the bread (Table 4), implying that the banana peel was the major contributory factor for enhancing the dietary fiber content of bread.

**TABLE 4:** Chemical composition (dry basis) of bread obtained from wheat flour mixed with different forms of green mature cooking bananas.

Type of sample	Sample formulation	Moisture (%)	Resistant starch (g/100 g)	Dietary fiber (%)
Fresh	T1	61.67 ± 0.40^a^	1.44 ± 0.15^ab^	1.95 ± 0.01^a^
	T2	60.40 ± 0.20^a^	1.59 ± 0.08^a^	1.52 ± 0.18^a^
Flour	T3	56.63 ± 0.03^b^	1.70 ± 0.04^a^	1.20 ± 0.25^ab^
	T4	57.50 ± 0.03^ab^	1.59 ± 0.08^a^	0.68 ± 0.13^b^
Control	C1	56.45 ± 0.10^b^	1.08 ± 0.01^b^	0.57 ± 0.05^b^
	C2	60.65 ± 0.20^a^	2.21 ± 0.01^a^	0.65 ± 0.12^b^

*Note:* Values in columns with similar superscript letters are not significantly different at *p* > 0.05. Values are means of two independent replicates.

Abbreviations: C_1_: 100% Wheat flour; C_2_: 50:50 (Banana flour: Wheat flour); T1: Fresh whole banana fingers; T2: Fresh banana pulp; T3: Whole banana finger flour; T4: Banana pulp flour.

### 3.4 Sensory Acceptability of the Wheat‐Banana Composite Bread

Table 5 indicates results of mean scores of sensory acceptability of the wheat‐banana composite bread. Generally, the addition of bananas caused a significant decrease in the sensory quality attributes of the composite bread compared to the 100% wheat alone sample (*p* < 0.05). The appearance scores for bread made from 100% wheat (C1) were significantly higher than that of the wheat‐banana composite. The mean scores for the color of the breads ranged from 4.88 to 7.80 with the 100% wheat control sample (C1) having the highest mean score while the 50% wheat flour: 50% banana had the lowest. Flavor and taste of the composite bread significantly decreased with the addition of bananas compared to the wheat alone sample (*p* < 0.05). However, the flavor score for T4 was not significantly different from that of C1. The bread texture softened with the addition of banana. However, bread made from banana pulp flour (T4) exhibited a texture that was not significantly different from that of the 100% wheat (C1) sample (*p* > 0.05). Mean scores for general acceptability ranged from 4.53 to 7.05 with the 100% wheat bread (C1) exhibiting the highest acceptability. Wheat‐banana composite bread had moderate acceptability, with scores ranging from 4.53 to 5.10 (Figure 1).

**TABLE 5:** Sensory attributes of bread obtained from wheat flour mixed with different forms of green mature cooking bananas.

Type of sample	Sample formulation	Appearance	Color	Flavor	Taste	Texture	General acceptability
Fresh	T1	4.58 ± 1.87^c^	5.03 ± 2.03^bc^	4.90 ± 2.13^b^	4.60 ± 1.85^b^	4.65 ± 2.05^b^	4.53 ± 2.11^b^
	T2	5.05 ± 1.87^bc^	5.45 ± 1.89^bc^	4.98 ± 2.07^b^	4.95 ± 1.84^b^	4.80 ± 1.90^b^	4.93 ± 1.90^b^
Flour	T3	6.15 ± 1.85^b^	5.73 ± 2.14^bc^	4.38 ± 1.75^b^	3.50 ± 2.17^c^	4.88 ± 2.34^b^	4.63 ± 2.05^b^
	T4	5.30 ± 2.04^bc^	5.20 ± 2.26^bc^	5.35 ± 1.87^ab^	4.98 ± 1.86^b^	5.25 ± 1.94^ab^	5.10 ± 1.96^b^
Controls	C1	7.90 ± 1.15^a^	7.80 ± 1.44^a^	6.45 ± 1.89^a^	6.45 ± 0.97^a^	6.48 ± 1.95^a^	7.05 ± 1.83^a^
	C2	5.03 ± 2.13^bc^	4.88 ± 2.17^c^	4.85 ± 1.94^b^	4.83 ± 2.18^b^	4.90 ± 2.20^b^	4.53 ± 2.03^b^

*Note:* Values in columns with similar superscript letters are not significantly different at *p* > 0.05; *n* = 30; Values are means of two independent replicates; means correspond to hedonic scale ratings of 1–9, where 1 = extremely dislike and 9 = extremely like.

Abbreviations: C_1_: 100% Wheat flour; C_2_: 50:50 (Banana flour: Wheat flour); T1: Fresh whole banana fingers; T2: Fresh banana pulp; T3: Whole banana finger flour; T4: Banana pulp flour.

## 4 Discussion

Optimum dough development is the result of hydration and uniform distribution of ingredients during mixing, following water absorption. Higher WA indicates higher amounts of water to be added to flour yielding a higher proportion of the dough, which is desirable for bread production. Lower indices could imply a lower bread volume. The WA indices of fresh banana samples were comparable to dry (flour) samples. Low baking strength is undesirable for bread making. The low indices of baking strength observed for banana‐wheat composite doughs could indicate weakening of the gluten structure by entrapment of the banana particles into the gluten network structure. Resistance to deformation (PrMax) is used to predict dough handling behavior and tolerance in the fermentation stage. All banana‐wheat composite doughs in this study had low PrMax values probably due to gluten dilution thereby reducing the extensibility of the bread dough. The reduced elasticity of the banana‐wheat composite dough could be explained by the fact that the non‐gluten chemical constituents in bananas do not readily bind with those of gluten to enhance dough elasticity thereby not enhancing the gluten network (Roccia et al. 2009; Maforimbo et al. 2007).

Bananas have a higher water retention capacity. The higher baking loss in composite flours could be attributed to a higher water absorption capacity of banana flour as compared to wheat flour. The low bread volume in samples formulated from whole fresh fingers could be attributed to gluten dilution, and mechanical disruption of the gluten network structure resulting into reduced dough elasticity (Paraskevopoulou et al. 2010; Doxastakis et al. 2002). The differences in bread volume could be linked to rheological properties of the dough, which are in turn, determined by differences in technological characteristics of bananas and wheat in terms of bread making. Similar to the findings of this study, Rahman et al. (2021) observed a decrease in bread volume with increasing banana flour. The addition of green bananas (with their peel) flour was also reported to decrease the specific volume of bread (Gomes et al. 2016). The increased crust and crumb browning in bread made from banana—wheat composite flour could have probably been due to Maillard reactions. Other studies have indicated that 5%–20% partial substitute of wheat with bananas resulted in improved color of both the crust and crumb of bread (Rahman et al. 2021; Gomes et al. 2016).

The high moisture content of fresh whole banana composite bread could be attributed to the presence of more soluble constituents such as starch and dietary fiber. Such substances have a higher affinity for water requiring less amount of water for hydration compared to the constituents in the dough system (Doxastakis et al. 2002; El‐Soukkary 2001). Similar to the findings of this study, compositing bananas with wheat significantly increased the moisture content of bread (Rahman et al. 2021; Gomes et al. 2016). Bananas enhanced the resistant starch content of bread. Noor et al. (2012) and Zhang et al. (2005) attributed the high levels of resistant starch in banana‐wheat composite bread to the high starch content in the green bananas (70%–80%, dry weight basis) and the presence of a high level of insoluble residues of dietary fiber in the banana flour. Banana starch has been reported to be particularly rich in resistant starch (Kembabazi et al. 2025). Addition of whole bananas resulted in bread with higher levels of dietary fiber. This is in line with the findings of Gomes et al. (2016) who reported that 10%–20% partial substitute of wheat flour with green bananas (with their peel) flour produced bread with higher fiber content. Bananas are a potential functional ingredient in bread due to their improved composition of functional ingredients such as resistant starch and dietary fiber (Ramli et al. 2010; Ovando‐Martinez et al. 2009). Dietary fiber buffers acid in the stomach, increases fecal softness, fecal bulk, water binding capacity, organic binding capacity, stimulates intestinal evacuation, reduces intestinal transit time, and binds diverse substances including cholesterol, all of which enhance the removal of stagnant or potentially detrimental materials from the bowels (Dhingra et al. 2012; Lattimer and Haub 2010). Thus, enriching wheat with other matrices such as bananas may be important to produce bread with high fiber content and enhanced health benefits (Petrontino et al. 2025).

Addition of bananas caused a significant decrease in the sensory quality attributes of bread. However, the wheat‐banana composite bread had moderate acceptability. In contrast to the findings of this study, Rahman et al. (2021) reported that compositing wheat flour with banana flour gave higher average score values for flavor, color, and overall acceptability. Noteworthy, Rahman et al. (2021) substitution levels (5%) were below the 30% substitution levels used in the current study. In another study, Gomes et al. (2016) showed that bread made from wheat flour composited with 20% green bananas (with their peel) flour had similar purchase intent and acceptability as traditional bread.

The correlations between rheological properties of the dough to the physical and sensory attributes of the bread were assessed using principal component analysis technique (PCA). In this study, PCA (Figure 5) was used to understand the factors that influence the rheology of the dough, physical and sensory attributes of the bread. The two principle components explained 62.26% variability; PC1 and PC2 accounted for 32.65% and 29.61% of the variables, respectively. Parameters that formed smaller angles between them indicated a positive correlation, while those closer to 90° or 180° indicated neutral or negative relationships, respectively. The first PC showed the largest possible variance in data and each succeeding component in turn had the highest variance possible under the constraint that it is orthogonal to the preceding components. The PC1 represents the effects of loaf weight, firmness, baking loss and moisture while PC2 represents the following textural parameters; elasticity, resilience, cohesiveness, specific volume, springiness, cross sectional area, tenacity to extensibility ratio as well as sensory attributes including color, appearance, general acceptability, flavor and taste. Cohesiveness, specific volume, springiness, Prmax, appearance, flavor, taste and texture were confined in the same quadrant indicating a positive relationship among them. Similarly, color, moisture and baking loss presented in the same quadrant depicting a positive relationship among them. Principal component analysis showed that physical or rheological properties of dough play a major role in bread quality.

**FIGURE 5:**
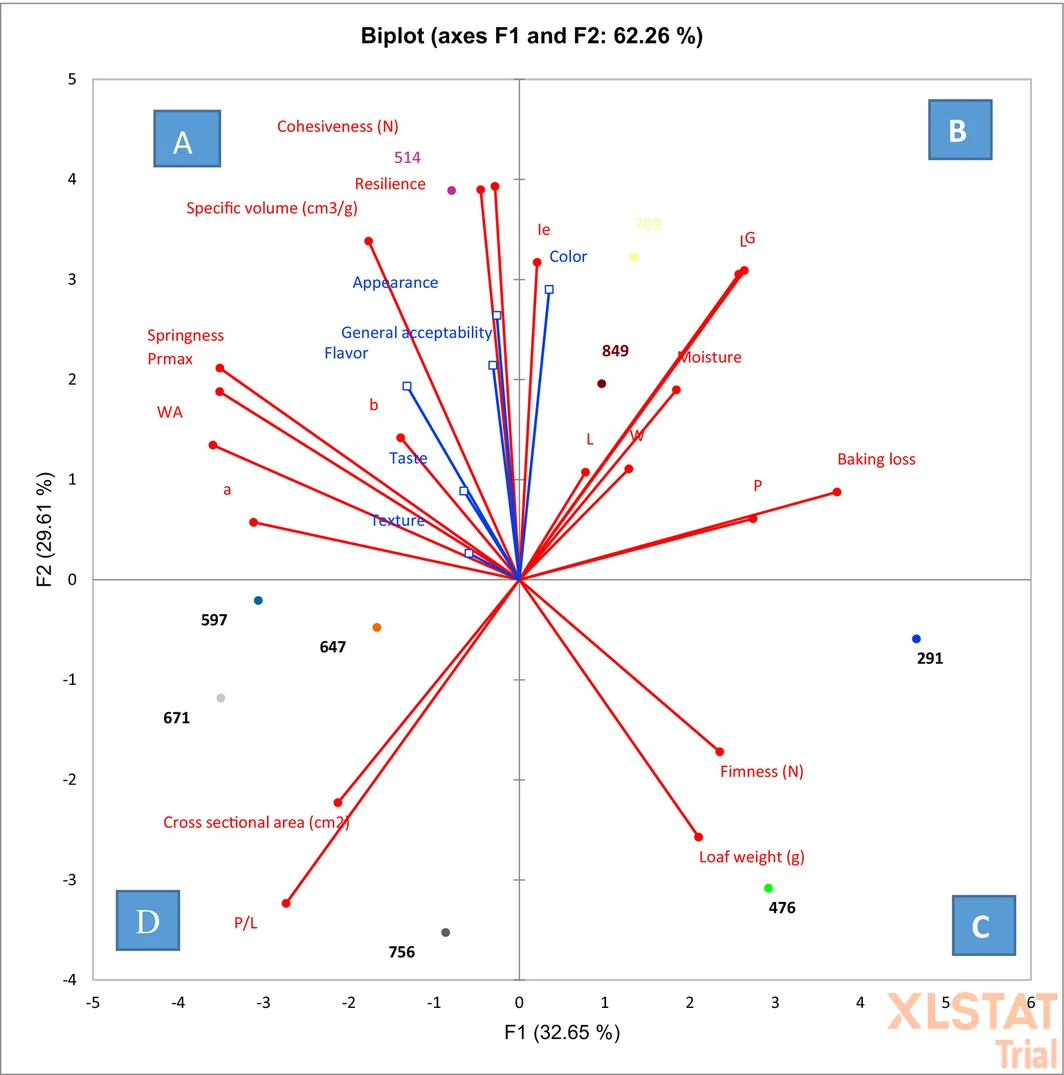
Principal component analysis of rheological, physical and sensory properties of bread obtained from wheat flour (70%) composited with different forms (fresh vs. flour, whole finger vs. pulp) of green mature cooking bananas (30%). P = peak height, L = dough extensibility, P/L = tenacity‐to‐extensibility ratio, Ie = elasticity index/elastic resistance, G = inflation required for maximum dough development, W = dough strength, PrMax. Bread samples: 597 = pulp flour, 647 = whole banana flour, 849 = fresh pulp.

## 5 Conclusions

This study evaluated the effect of 30% substitution of wheat flour with bananas (fresh whole fingers, fresh pulp, whole finger flour, and pulp flour) on rheological, physico‐chemical, and sensory properties of bread. Addition of fresh and/or powdered green mature cooking bananas increased resistant starch and dietary fiber content of bread. It increased the time required to reach maximum consistency of the dough but had minimal effect on water absorption capacity. Addition of bananas reduced dough resistance to deformation and baking strength during handling and fermentation. Whereas partial substitution of wheat with fresh whole green banana fingers or fresh banana pulp had relatively similar alveograph profiles to those of the 100% wheat flour implying the possibility of utilizing fresh bananas in bread dough making without investing resources in drying and milling, higher substitution levels may reduce bread volume, sensory acceptability, and dough strength. Lower levels of banana substitution may benefit bread quality.

## Author Contributions

**Ediriisa Mugampoza:** conceptualization, investigation, writing – original draft, funding acquisition, methodology, validation, visualization, writing – review and editing, software, formal analysis, project administration, data curation, supervision, resources. **Norah Nankya:** conceptualization, investigation, writing – original draft, methodology, validation, visualization, writing – review and editing, software, formal analysis, data curation. **Arthur Tabula:** conceptualization, investigation, writing – original draft, methodology, validation, visualization, writing – review and editing, software, formal analysis, data curation. **Martin Mutambuka:** conceptualization, investigation, funding acquisition, writing – original draft, methodology, validation, visualization, writing – review and editing, software, formal analysis, project administration, data curation, supervision, resources. **Khadijah Nakyinsige:** conceptualization, investigation, funding acquisition, writing – original draft, methodology, validation, visualization, writing – review and editing, software, formal analysis, project administration, data curation, supervision, resources.

## Conflicts of Interest

The authors declare no conflicts of interest.

## Data Availability

The data that support the findings of this study are available from the corresponding author upon reasonable request.
